# The National Insurance Bill and Hospital Benefit

**Published:** 1911-12-09

**Authors:** 


### THE NATIONAL INSURANCE BILL AND HOSPITAL BENEFIT.

### How to Prepare for a Perfected Hospital and Health Service for Great Britain.

Further and closer investigation of the sources of income of the voluntary hospitals proves to demonstration on the actual figures that, when legacies are taken into consideration, an auditor and arbitrator must find, if he be a just man, that the damage to the voluntary hospitals under the Bill as it stands will, in all probability, not be less than a sum equal to 50 per cent. of their present income from voluntary sources, including legacies. That is the case of the voluntary hospitals *vis-à-vis* with the Government in regard to this legislation, as we are prepared to demonstrate by actual figures when desired.

### The Insured and Hospital Benefit.

A closer study of the Bill and the speeches made by the Chancellor of the Exchequer demonstrates a new and important point which on the evidence renders it essential, if the Bill is to succeed, that certain clauses shall be amended so as to guarantee, where necessary, the provision of hospital benefit to those of the assured who will require it in the ordinary course. In order to understand this point clearly it is essential to remember three facts: First, the German scheme, which is avowedly the basis upon which the National Insurance Bill has been built up, guarantees to ensure a certain and sufficient relief in case of illness during a fixed number of weeks to each insured person. In Germany there are practically no voluntary hospitals in the English sense, and hospital provision already exists upon a plan whereby the State can secure on a *pro rata* payment the hospital provision required by each of the insured to ensure, when occasion arises, all the hospital care which they may need.

### Promises Broken and Unfulfilled.

Secondly, when the Chancellor of the Exchequer introduced the Bill into the House of Commons, he laid it down that it would provide free medical relief for every insured person with no taint of charity. Now, unless the Bill contains power to secure hospital accommodation for the insured, when necessary, it will be impossible for the pledge of the Chancellor of the Exchequer just referred to to be fulfilled, because the admission of an insured person without payment to a voluntary hospital must constitute charitable relief.

Again, in his speech at Birmingham on June 10, 1911, the Chancellor said: "The first thing to do in our Bill is to provide adequate medical treatment for every workman in the United Kingdom." Adequate medical treatment must include hospital treatment, for experience shows that it is essential in all urban communities, in order to meet the medical needs of the population, that hospital accommodation shall be provided to the extent of two beds for each thousand of the population. The Bill, according to the Chancellor, will create 15,000,000 insured persons who are **guaranteed** by the Government, in virtue of the payments made by themselves, their employers, and the State, **adequate medical treatment in every case.** It follows that this guarantee cannot exist, in fact, unless the Bill makes arrangements whereby such hospital provision will be readily obtainable. The voluntary hospitals under their constitution exist for the accommodation of the poorer members of society, including the working classes, where the breadwinner, though able to maintain himself and his family when well, cannot defray the cost of hospital treatment for himself and the members of his family in the days of accident or disease. **It is clear, therefore, that no voluntary hospital could admit an assured person, who is guaranteed by the Bill adequate medical treatment, by placing him in a free bed without cost to the Government or the assured. If they did this, the public who support the voluntary hospitals, and who have to provide by direct payment for the guaranteed medical treatment under the Bill of the persons they employ, must refuse in self-defence to continue their subscription to the voluntary hospitals.**

### The Case of London.

Take the case of London as an example. The population of Greater London, including the Metropolitan and City Police districts, according to the 1911 census, is approximately 7,252,963. The assured will represent (on the basis of the figures of the 1901 census, those for 1911 not being yet available) 36 per cent. of this population, or approximately 2,500,000 assured persons. To meet the adequate medical treatment claimed by Mr. Lloyd George under the Insurance Bill of these insured people in London alone, it is essential that not fewer than 5,000 hospital beds shall be available for their sole use. The London voluntary and endowed hospitals have collectively 10,500 beds, of which 10,000, on an average, are constantly occupied. If, therefore, as we believe will be found to be the case in a few years, the revenue of these hospitals from voluntary sources is adversely affected to the extent of 40 per cent., there will only be enough money left to the London voluntary hospitals to maintain about 5,000 hospital beds, or the number which approximately will be required for the sole use of insured presons under the National Insurance Bill. Put in this simple way, we hoped that the Chancellor of the Exchequer would agree that it is essential for the Bill to make provision for hospital benefit for the insured under it, and the pressing question to determine, now, is: how can this be done in the simplest and readiest manner at the smallest possible cost?

### A Ready and Relatively Inexpensive Means of Providing Hospital Benefit.

The **suggested amendments** to the Bill are as follows:—

Clause 10, section (1) (*b*): treatment in hospitals in addition to sanatoria, accidents in addition to disease, and institutional benefit are included.

Clause 12, section (1), line 5: institutional benefit is substituted for sanatorium benefit, and also in line 9.

Clause 14, section (2) (*b*), line 1: provision is made for the addition of the word "hospital" after the word "sanatorium"; and in the same clause, section (*c*), line 2, after the word "inmate," the following words are added: "otherwise than as a person in receipt of institutional benefit."

Clause 15, section (1), line 5, "institutional" is substituted for "sanatorium" benefits.

Clause 17, section (1), line 1, "institutional" is substituted for "sanatorium" benefit; and in (1) (*a*), line 2, after the words "such disease" the words "or accident," and line 3, after the word "sanatoria" the word "hospitals" are inserted. In line 5 (1) (*a*), after the word "sanatoria" the word "hospitals" is inserted. In the same clause, section (1) (*b*), line 2, the word "hospitals" is inserted after the word "sanatoria." In the same clause, section (2), line 2, the word "sanatorium" is struck out and the word "institutional" is inserted. In section (3) of the same clause the word "sanatorium" is struck out and the word "institutional" inserted. At the end of this section the following words are added: "Provided that, where a duly qualified member of the medical staff of any such hospital as aforesaid certifies that an insured person is in urgent need of admission to such hospital, such certificate shall have the same validity, so far as regards the admission and subsequent treatment of such person, as a recommendation by the local Health Committee."

In the same clause, section (4), line 2, substitute the word "institutional" for the word "sanatorium," and in line 4 insert the word "hospital" after the words "any sanatorium."

Clause 20, lines 3 and 4, after the words "think fit," delete the following words "to hospitals and other charitable institutions, or."

These amendments, if agreed to, would make provision for hospital treatment upon a basis which will enable the cost of such provision to be reduced to a minimum, by agreement through the Insurance Commissioners with each voluntary hospital to set aside certain wards and beds for the reception and treatment of insured persons for a *pro rata* payment representing the actual cost of such treatment to the hospitals, plus 10 per cent. to cover all the medical and surgical treatment each case may require. How is the money for this *pro rata* payment to be provided? As the amendments in the Bill would indicate, the sum set aside for sanatorium benefit must be made available as to a certain proportion for hospital benefit, an arrangement which will have the advantage of securing that all expenditure under both heads shall be made with the utmost care, so as to avoid hasty expenditure upon buildings and sanatoria provision, and the undue multiplication of such buildings and provision, which may, and probably will, otherwise take place. After all, sanatoria are not a cure but merely one of several palliatives which, collectively, are already proving that it is possible practically to eradicate tuberculosis, in the same way that typhus fever, small-pox, typhoid, scarlet fever, and other pests of our population have been successfully grappled with.

### Sanatoria Waste Can Be Avoided.

To succeed, so far as medical treatment is concerned, the Insurance Bill should be so drawn as to provide (as far as possible) means which will simplify the problem of Poor-Law reform when it is tackled by the Government, so that overlapping and many contingent and avoidable evils of the existing want of co-operation and inter-communication may be absolutely prevented for the future. Take the case of sanatoria buildings, bearing in mind that local authorities all over the country largely regard such buildings as being required for cases of advanced tuberculosis—that is, those who have passed from the first stage of the disease into later stages, where the hope of recovery is greatly diminished or altogether impossible. There is at the present time in the United Kingdom a large number of buildings connected with local, Poor-Law, and health authorities which at a very small cost may readily be converted into sanatoria for the reception of this large class of tuberculosis patients. The recognition of this fact at the commencement must tend materially to reduce the cost of providing sanatoria, and also the cost of Poor-Law relief under a Poor-Law Reform Bill.

### An Economical Point.

Again, as the estimates of the cost of bringing in the system of national insurance under Mr. Lloyd George's Bill, and the form in which they are presented, demonstrate, the charges and cost of this legislation will take a few years to arrive at, and it would therefore be possible, under a matured and careful scheme like that here suggested for hospital benefit, gradually to cover the whole ground of sanatoria and hospital accommodation out of the funds provided under the Bill as it stands at present, at any rate in the first two or possibly more years of its working. So the plan here outlined is one which must prove economical, in the sense that it will operate as a means of enforcing the strictest care in the expenditure of money upon such provision, directly in the way of buildings, and indirectly in the way of benefit, whilst it guarantees in the most effectual way possible that all such provision shall be the best, though least costly, and most adequate for the purpose.

### Increased Hospital Accommodation.

There remains the question of increased hospital accommodation. Some years ago—believing, as we do, that the solution of our hospital problem lies in an adaptation of the Continental system, whereby each hospital is organised in departments: (*a*) for cases sent in by the Government or municipalities which pay the actual cost of the treatment given to the patients; (*b*) provision for those patients who can pay a portion, though not the whole, of the actual cost of treatment; (*c*) those who can pay the whole cost of their treatment; and (*d*) those who can pay the whole cost of their treatment and accommodation when in the hospital, together with the medical fees and charges—we found, as the arrangements made by the medical profession with the working classes in Birmingham prove, that it is possible to arrange with the medical staff or consultants in connection with the hospitals for reduced fees in the case of artisans, workers and others, whose means are strictly limited. We further found that a large number of hospitals have sites available in connection with their present establishments, whereon pavilions can be readily erected at a minimum cost, where patients, of one or all of the four classes just referred to, can be treated under the best conditions at a minimum cost. Several of these hospital authorities were willing to grant the sites for the erection of such pavilions thereon, and to undertake the administration of these new sections of the hospital upon terms to be mutually agreed. Most, therefore, of the accommodation which may be required can be readily obtained at a minimum cost, provided the capital sum required to defray the cost of erecting the new buildings or pavilions for the treatment of patients is forthcoming. Our view is that existing pavilions should be utilised as far as possible, by an arrangement between the hospital authorities and the Insurance Commissioners to pay *pro rata* the cost actually incurred by the hospital in the treatment of insured or State cases, plus 10 per cent. Where it is necessary to provide further hospital pavilions, our suggestion is that the Government should agree to empower the department of the Government charged with health and hospital matters, with the consent of the Treasury, to make loans to such hospital convalescent home or infirmary at such rate of interest, not being less than 3 per cent. per annum, as may be arranged, and that every such loan shall be made repayable by a sinking fund. All sums payable in respect of the interest and sinking funds on any such loan to constitute a first charge upon any sums payable to the hospital, convalescent home, or infirmary in respect of any institutional benefit under the National Insurance Bill. In order to effect this arrangement, which will enable the Government, without further addition to the Budget or any increase of the National Debt, to effect this invaluable reform and benefit all classes of the community in regard to the treatment of illness and accident, we have had drafted and now submit a new sub-section of clause 60, being sub-section (5), which we give below.

### The Solution of Many Difficulties.

It was hoped that this relatively brief statement of a solution of many pressing problems would commend itself to the Chancellor of the Exchequer, for it contains the means of making the scheme of national insurance statesmanlike and complete, and certain to prove adequate and to work satisfactorily and economically when the Bill becomes an Act of Parliament. The National Insurance Bill would then, with the momentous changes Mr. Lloyd George has introduced, form a perfected and material step towards Poor Law reform, and make the task of the Government in framing a Poor Law Reform Bill relatively easy, so that the two enactments should give to the United Kingdom the most perfect system of national health provision to be met with in any part of the world.

### Suggested New Sub-section to Clause 60, being Sub-section (5).

60 (5). In order to provide such additional accommodation in any hospital, convalescent home, or infirmary approved by the Local Government Board as may be required for the purposes of institutional benefit under this Act, the Local Government Board may from time to time, with the consent of the Treasury, make loans to such hospital, convalescent home, or infirmary, at such rate of interest not being less than 3 per cent. per annum as may be arranged, but every such loan shall be made repayable by means of a sinking fund in not more than — years, and all sums payable in respect of the interest and sinking fund on any such loan shall constitute a first charge upon any sums payable to the hospital, convalescent home, or infirmary, in respect of any institutional benefit under this Act, and also upon any sums paid or payable to the hospital, convalescent home, or infirmary by or in respect of any individual receiving treatment in any building erected wholly or partly out of moneys provided under this sub-section.

### The Position To-day and Work for To-morrow.

So far as we can gather, the fight for the inclusion of hospital benefit in the Insurance Bill dealt with in the foregoing article is only adjourned, not concluded. Having regard to the want of time owing to the rushing of this Bill through Parliament, it was determined, with, we understand, great reluctance in many quarters, to suffer the question of the inclusion of hospital benefit to stand over owing to the special difficulty of the circumstances of the moment. There was a general feeling that when the Bill was passed, if the contentions advanced in the foregoing exposition were sound, there would be an uprising of public opinion, led by the working classes and supported by the voluntary hospitals throughout the country, which would bring such forces to bear upon members of Parliament that time would be found to pass a short amending Act, relieving the working classes from the taint of charity with which they are threatened, and providing all the hospital benefit the insured will need free from any taint of charity. That was the boast of the Chancellor of the Exchequer when he introduced the Bill. He insisted upon the same principle, with pride and assurance, at the great meeting he addressed at Birmingham in June, and he must have had the same thing in his mind when he bluntly told the voluntary hospital managers that they had a remedy against financial loss due to the Bill by refusing treatment to insured persons who were ineligible for free medical relief. What is wanted is a number of trained and experienced writers, speakers, and workers in all situations of life to join hands in the consolidated effort which must now be made to remove the wrongs with which the hospitals and British workmen are threatened under this Bill.

